# A Human Induced Pluripotent Stem Cell-Derived Isogenic Model of Huntington’s Disease Based on Neuronal Cells Has Several Relevant Phenotypic Abnormalities

**DOI:** 10.3390/jpm10040215

**Published:** 2020-11-09

**Authors:** Tuyana Malankhanova, Lyubov Suldina, Elena Grigor’eva, Sergey Medvedev, Julia Minina, Ksenia Morozova, Elena Kiseleva, Suren Zakian, Anastasia Malakhova

**Affiliations:** Federal Research Center Institute of Cytology and Genetics, Siberian Branch of Russian Academy of Sciences, 630090 Novosibirsk, Russia; suldinalubov@gmail.com (L.S.); evlena@bionet.nsc.ru (E.G.); medvedev@bionet.nsc.ru (S.M.); minina_jul@bionet.nsc.ru (J.M.); morozova.kn@gmail.com (K.M.); elka@bionet.nsc.ru (E.K.); zakian@bionet.nsc.ru (S.Z.); amal@bionet.nsc.ru (A.M.)

**Keywords:** Huntington’s disease modeling, genome editing, induced pluripotent stem cells

## Abstract

Huntington’s disease (HD) is a severe neurodegenerative disorder caused by a CAG triplet expansion in the first exon of the *HTT* gene. Here we report the introduction of an HD mutation into the genome of healthy human embryonic fibroblasts through CRISPR/Cas9-mediated homologous recombination. We verified the specificity of the created *HTT*-editing system and confirmed the absence of undesirable genomic modifications at off-target sites. We showed that both mutant and control isogenic induced pluripotent stem cells (iPSCs) derived by reprogramming of the fibroblast clones can be differentiated into striatal medium spiny neurons. We next demonstrated phenotypic abnormalities in the mutant iPSC-derived neural cells, including impaired neural rosette formation and increased sensitivity to growth factor withdrawal. Moreover, using electron microscopic analysis, we detected a series of ultrastructural defects in the mutant neurons, which did not contain huntingtin aggregates, suggesting that these defects appear early in HD development. Thus, our study describes creation of a new isogenic iPSC-based cell system that models HD and recapitulates HD-specific disturbances in the mutant cells, including some ultrastructural features implemented for the first time.

## 1. Introduction

Huntington’s disease (HD) is an actively investigated neurodegenerative disorder caused by an expansion of CAG triplets (>36) in the first exon of the *HTT* gene encoding the huntingtin protein. Mutant huntingtin contains an elongated polyglutamine tract, which changes the protein conformation thereby leading to the loss of normal huntingtin function and a toxic effect on striatal medium spiny neurons (MSNs). Moreover, there is a direct correlation between the number of CAG triplets and the age of onset of the first HD symptoms. Loss of MSNs causes motor dysfunctions, cognitive impairment, and ultimately, patient death [1].

To study the molecular mechanisms of HD, relevant model systems are needed that accurately reproduce the phenotype of the disease. Advances in the technology for generation of induced pluripotent stem cells (iPSCs) have opened up new possibilities for modeling of diseases because it is now possible to create cell models from patients’ cells [2]. Nonetheless, single-nucleotide polymorphisms in the genomes of different patients can strongly affect the research results. A solution to this problem is the creation of isogenic cell lines [3]. The latter have an identical genetic background and differ from each other only by the disease-causing mutation. Genome-editing tools such as the CRISPR/Cas9 (clustered regularly interspaced short palindromic repeats/CRISPR-associated protein 9) system can be used to create isogenic cell lines. CRISPR/Cas9 allows for efficient and specific modification of the cell genome. An isogenic pair of cell lines can be obtained in two ways: the first method is to correct the mutation in patient-specific cells, and the second one is to introduce the mutation into “healthy” cells. Isogenic cell models are promising platforms for drug screening and for research on the molecular pathogenesis of HD.

In 2012, the first HD isogenic cell lines were obtained by correction of the mutation in patient-specific cells via homologous recombination [4]. The resultant iPSCs were differentiated into MSNs in vitro and in vivo. The correction of the mutation normalized the signaling pathways disturbed in HD (TGF-β, cadherin, activation of caspases, and brain-derived neurotrophic factor (BDNF)) and increased survival, and restored mitochondrial energy production of the neural stem cells obtained from iPSCs.

The CRISPR/Cas9 system was used to introduce the pathogenic mutation into *HTT* in 2014 [5]. The researchers employed a plasmid vector containing 97 CAG repeats and a neomycin resistance gene for rapid selection of recombinant clones as a donor template for homologous recombination. To confirm the expression of the mutant huntingtin, the authors performed screening based on Western blot analysis with antibodies that bind to the polyglutamine tract containing more than 38 glutamine residues. This method of introducing a mutation is efficient; however, the presence of a selection cassette may have an undesirable background effect on the studies’ results.

Researchers from Singapore used an alternative strategy based on the correction of the HD mutation in 2017 [6]. In that work, they utilized the Cas9 nickase that introduces single-stranded DNA breaks, to reduce off-target activity and to increase the efficiency of homologous recombination [7]. The donor plasmid contained the piggyBac transposon selection cassette, which enables seamless removal of the selection cassette using transposase. The selection cassette also contained a puromycin resistance gene for positive selection and a herpes simplex virus thymidine kinase gene for negative selection. The mutant cells and cells with the corrected mutation were differentiated into forebrain neurons. The cells with the mutation had phenotypic abnormalities, such as low efficiency of formation of neural rosettes, high sensitivity to the withdrawal of growth factors, and impaired mitochondrial respiration. Nonetheless, all these disturbances were not observed in the isogenic corrected cells. Moreover, a comparative analysis of the transcriptome of the cells carrying the mutation and an isogenic control—as well as a non-isogenic control derived from a “healthy” donor—uncovered many gene expression differences between the mutant cells and the non-isogenic “healthy” control, while such differences were not found in a comparison with the isogenic “healthy” control. Thus, the genetic background affected the differential background expression of genes thus confirming the importance and necessity of an isogenic control.

In 2019, the same authors created a panel of isogenic cell lines based on human embryonic stem cells [8]. They introduced an expanded CAG tract of various lengths into *HTT* of the cells; this approach helped them to model HD of various severity levels. Then, the cells were differentiated into various cell types including neurons, hepatocytes, and muscle cells. Transcriptomic and proteomic analyses of the resultant cell types revealed differential susceptibility of the tissues and cell types to HD pathology.

Here, we used a system based on CRISPR/Cas9 and homologous recombination to introduce an expanded CAG tract (69 CAG) into the first exon of the *HTT* gene in human embryonic fibroblasts. The donor vector for homologous recombination did not contain any selection cassette. The cassette-free approach ensured only CAG-dependent differences between the mutant and control cells. Cell clones with the expanded CAG tract in *HTT* were reprogrammed into a pluripotent state by means of episomal vectors, and their pluripotent status was described in detail. The mutant iPSCs and a “healthy” isogenic control as well as a non-isogenic mutant control were differentiated into MSNs, which are most affected in HD. In this work, it was important to show that the specific neural derivatives of cells with expanded CAG triplets in *HTT* have a mutant phenotype as the non-isogenic mutant control compared to the “healthy” isogenic control. Evaluation of the mutant cell lines revealed a number of phenotypic abnormalities that were also found in patient-specific cells. Our study confirms that the obtained isogenic model system is useful for research into the molecular basis of HD and for screening of drug candidates without the need to consider the influence of the genetic background.

## 2. Materials and Methods

### 2.1. Generation of the HTT-Mutant Isogenic Cell Clones

The isogenic cell clones were generated from human embryonic fibroblasts (named MA№1) via editing of the genome with the CRISPR/Cas9 system [9,10]. The fibroblasts were cultivated in a medium consisting of Dulbecco’s modified Eagle’s medium (DMEM)/F12 (1:1; Thermo Fisher Scientific, Waltham, MA, USA), 10% of fetal bovine serum (Thermo Fisher Scientific, Waltham, MA, USA), 200 mM GlutaMAX (Thermo Fisher Scientific, Waltham, MA, USA), and 100 U/mL penicillin with 100 µg/mL streptomycin (pen/strep; Thermo Fisher Scientific, Waltham, MA, USA). We designed a 22 nt single guide RNA sequence (5′-GAAGGCCTTCATCAGCTTTTCC-3′) to cut the genomic DNA 20 bases upstream of the CAG repeat tract in exon 1 of *HTT* and inserted it into the pSpCas9(BB)-2A-GFP (pX458) plasmid (Addgene, Watertown, MA, USA). Cells were cotransfected with the CRISPR/Cas9-encoding plasmid and donor construct using polyethylenimine (Santa Cruz Biotechnology, Dallas, TX, USA). The presence of the EGFP marker gene in the pX458 plasmid allowed the transfected cells on Cell Sorter S3e to be sorted (Bio-Rad, Hercules, CA, USA). Thus, EGFP-positive single-cell clones were obtained. Genomic DNA was isolated from the cell clones growing in 96-well plates using the Quick-DNA 96 Kit (Zymo Research, Irvine, CA, USA). The length of the mutant *HTT* allele was analyzed by PCR [65 mM Tris-HCl pH 8.9, 16 mM (NH_4_)_2_SO_4_, 1.5 mM MgCl_2_, 0.05% Tween 20, 3% glycerol, 6% dimethyl sulfoxide (DMSO), 0.5 U of Taq polymerase, primers 0.2 μM each, and 50–200 ng of genomic DNA] on a C1000 Touch Thermal Cycler (Bio-Rad, Hercules, CA, USA) (96 °C for 5 min; then 40 cycles of 96 °C for 20 s and 72 °C for 3 min; with final extension at 72 °C for 15 min) using primers HTT-F 5′-CCCAAGGCCACCTCGGCTCAGAGTC-3′ and HTT-R 5′-CGCAGGCTGCAGGGTTACCGCCATC-3′.

Cell lysates for Western blotting were prepared in RIPA buffer (Thermo Fisher Scientific, Waltham, MA, USA). Protein concentrations were determined by the BCA Protein Assay (Thermo Fisher Scientific, Waltham, MA, USA). Lysates (40 μg) were prepared in 4× SDS-PAGE loading buffer (200 mM Tris-HCl pH 6.8, 400 mM dithiothreitol, 8% sodium dodecyl sulfate (SDS), 0.4% bromophenol blue, and 40% glycerol). The samples were boiled for 5 min at 98 °C and separated by one-dimensional SDS-PAGE on a 7.5% polyacrylamide gel at 100 V for 4 h in running buffer (25 mM Tris base, 3.5 mM SDS, and 200 mM glycine) on ice. Overnight transfer was performed at constant voltage 30 V for 16 h onto an Immun-Blot PVDF membrane (Bio-Rad, Hercules, CA, USA) in transfer buffer (25 mM Tris base, 192 mM glycine, and 10% ethanol) at 4 °C. After blockage in 5% milk in tris-buffered saline supplemented with 0.05% Tween 20, a primary antibody [Anti-Huntingtin N terminus (3–16), H7540 Sigma, USA, dilution: 1:4000; or anti-SMC1 antibody, A300-055A Bethyl, USA, 1:4000] was incubated with the membranes overnight at 4 °C. The membranes were next incubated with a secondary antibody (an HRP-conjugated anti-rabbit IgG antibody; 1:10,000, Jackson ImmunoResearch, West Grove, PA, USA) at room temperature (RT) for 2 h in the blocking solution. Protein bands were detected by means of chemiluminescence with the Pierce ECL Western Blotting Substrate (Thermo Fisher Scientific, Waltham, MA, USA).

### 2.2. Analysis of CRISPR/Cas9-HTT Off-Target Activity

DNA was amplified in a PCR mixture [2×BioMaster HS-Taq PCR-Color (Biolabmix, Novosibirsk, Russia), primers 0.2 μM each, 50–100 ng genomic DNA] on the C1000 Touch Thermal Cycler (Bio-Rad) (95 °C for 5 min; then 35 cycles of 95 °C for 20 s, 65 °C for 20 s, and 72 °C for 30 s; with final extension at 72 °C for 5 min). The following primers were used: OFF-1 5′-TTGATTGGTGCCACTGTGTT/CACATGCAGGACAAAGGAGA-3′, OFF-2 5′-CACGATAAAACCCCCTCAAA/TCCAGCCAAATTGACTCTCC-3′, OFF-3 5′-CCCCTAACTTGGTGTGTGGT/CTGGTCCTTTGCTGACTGTG-3′, OFF-4 5′-CAAGACCAGGCATCTTCACA/ATGACTGGGGATCCAGCTTT-3′, OFF-5 5′-CGCAGTGCACCTGAGTGATA/GCAGCTTATGGAAAGGAGGA-3′, OFF-27 5′-CTTCTGAGCTTGAGCATCCA/TGGAGGAGTGGAGGAGAGAA-3′. The PCR products were sequenced by means of the forward primers. Sequence alignment was performed on benchling.com.

### 2.3. Genotyping of the CAG Expansion in Exon 1 of the HTT Gene by Fragment Analysis

DNA was amplified in the following PCR mixture: Colorless GoTaq Flexi Reaction Buffer (Promega, Madison, WI, USA), 2.5 mM MgCl_2_, 0.2 mM dNTP, 10% of DMSO, 0.5 U/μL Taq DNA polymerase, 0.5 pmol/μL FAM-labeled Forward primer [(Fam)5′-TGGCGACCCTGGAAAAGCTGAT-3′] and 0.5 pmol/μL Reverse primer (5′-GGTGGCGGCTGTTGCTGCTGCTG-3′). The reactions were run on thermocycler C1000 (Bio-Rad) with the following thermal cycling program: 95 °C for 5 min; then 35 cycles of 95 °C for 15 s, 63 °C for 15 s, and 72 °C for 25 s; with final extension at 72 °C for 25 min. Capillary electrophoresis was performed using capillary genetic analyzer ABI Prism 3130 (Applied Biosystems, Waltham, MA, USA), with size standard Liz 600; the obtained data were analyzed in Data Collection software v3.0 and GeneMapper v.4.0 (Applied Biosystems, Waltham, MA, USA).

### 2.4. Reprogramming of Human Embryonic Fibroblasts

Human embryonic fibroblasts (3 × 10^5^) were transfected with a set of episomal vectors encoding the Yamanaka factors: OCT4, KLF4, L-MYC, SOX2, LIN28, and Trp53 (500 ng of each vector; Addgene IDs #41855–58 and #41813–14) using a Neon Transfection System (Thermo Fisher Scientific, Waltham, MA, USA; the program for iPSC reprogramming: fibroblasts). The cells were cultured in DMEM/F12 supplemented with 10% fetal bovine serum, 1 mM GlutaMAX-I, pen/strep (Thermo Fisher Scientific, Waltham, MA, USA), and 2 ng/mL bFGF (StemRD, Burlingame, CA, USA). Starting from the 6th day, the cells were seeded onto mitotically inactivated mouse embryonic fibroblasts in KnockOut DMEM (Thermo Fisher Scientific, Waltham, MA, USA) with 15% knockout serum replacement (Thermo Fisher Scientific, Waltham, MA, USA), 0.1 mM non-essential amino acids (Thermo Fisher Scientific, Waltham, MA, USA), 2-mercaptoethanol (Sigma), pen/strep, 1 mM GlutaMAX-I, and 10 ng/mL bFGF. The primary iPSC colonies were picked manually and cultured under the conditions stated above. iPSC lines were dissociated with TrypLE (Thermo Fisher Scientific, Waltham, MA, USA) and split at 1:10 twice a week. The cells were cultured at 37 °C and 5% CO_2_.

### 2.5. iPSC Differentiation into MSNs

IPSCs were differentiated into MSNs according to a previously published protocol [10]. For terminal differentiation into MSNs, the precursor cells were plated onto the Matrigel-ESQ matrix at a density of 1–2 × 10^4^ cells/cm^2^; the maturation duration was 3 weeks.

### 2.6. Differentiation of iPSCs into Neural Rosettes

When iPSCs reached 90% confluence in 100 mm Petri dishes, the cells were detached with collagenase IV (working concentration 1 μg/mL, Sigma). The detached cells were gently washed with any medium and centrifuged for 5 min at 100× g. The supernatant was aspirated with a serological pipette, and the cell pellet was carefully resuspended in the iPSC medium without bFGF and transferred into Petri dishes coated with 1% agarose. The medium was refreshed every other day. On the 6th day of cultivation, the medium was changed to a neural differentiation medium consisting of DMEM/F12, 1× serum-free supplement N2 (Thermo Fisher Scientific, Waltham, MA, USA), 0.1 mM non-essential amino acids, pen/strep, and 5 ng/mL bFGF. On the 16th day, embryoid bodies were placed into Matrigel-ESQ matrix-coated plates and cultured for another 5–7 days. The cells were fixed and immunostained with antibodies against ZO-1 and MAP2 (Cell Signaling Technology, Danvers, MA, USA). The ImageJ freeware (version 1.53a, NIH, Bethesda, MD, USA; ImageJ. Available online: https://imagej.nih.gov/ij/ (accessed on 5 November 2020)) was utilized for analysis of three biological replicates. At least six random fields of view were analyzed.

### 2.7. Reverse-Transcription Quantitative PCR (RT-qPCR)

Total RNA was isolated with TRIzol (Thermo Fisher Scientific, Waltham, MA, USA). Reverse transcription of 1 μg of total RNA was performed with SuperScript III Reverse Transcriptase (Thermo Fisher Scientific, Waltham, MA, USA). RT-qPCRs were run on a LightCycler 480 Real-Time PCR System (Roche, Basel, Switzerland) with BioMaster HS-qPCR SYBR Blue 2× (Biolabmix, Novosibirsk, Russia). C_t_ values were normalized to the *ACTB* and *HPRT* genes by the ΔΔC_t_ method. For the list of primers used for quantitative reverse transcription PCR (RT-qPCR), see Table 1.

### 2.8. Immunofluorescence Staining

iPSCs were fixed in 4% paraformaldehyde for 10 min at RT, permeabilized in 0.5% Triton X-100 (Sigma, St. Louis, MO, USA) for 30 min at RT, and then incubated with blocking buffer (1% bovine serum albumin (BSA) in PBS; Sigma, St. Louis, MO, USA) for 30 min at RT. Primary antibodies were incubated with the cells overnight at 4 °C (Table 2). Secondary antibodies were added for 1.5–2.0 h incubation at RT (Table 2). All the antibodies were diluted in PBS supplemented with 1% BSA. Nuclei were counterstained with 4′,6-diamidino-2-phenylindole (DAPI; Thermo Fisher Scientific, Waltham, MA, USA). Micrographs were captured using a Nikon eclipse Ti-E microscope (Nikon, Tokyo, Japan) and NIS Elements software.

### 2.9. A Growth Factor Withdrawal Assay

MSN progenitors were seeded in a 48-well plate at density 1.7 × 10^5^ cells/cm^2^ in the NeuroB medium. Twenty-four hours after the plating, the medium was changed to Neurobasal (Thermo Fisher Scientific, Waltham, MA, USA) supplemented with 1% BSA (Sigma) and pen/strep. After 24 h, the cells were fixed and immunostained with an antibody against activated caspase 3 (Cell Signaling Technology, Danvers, MA, USA). The ImageJ software (version 1.53a, NIH, Bethesda, MD, USA) was employed for analysis of three biological replicates. At least six random fields of view were analyzed.

### 2.10. Statistical Analysis

Experiments intended to determine the relative gene expression level and neural-rosette differentiation and the assay of the sensitivity of progenitor cells to BDNF withdrawal were performed in three biological replicates. The measurements of neural-rosette size and caspase-3-positive cells were carried out in the ImageJ software (version 1.53a, NIH, Bethesda, MD, USA). Significance of differences in the compared mean values was verified by one-way ANOVA followed by Fisher’s test in the SPSS Version 11.0 statistical software package (IBM Corp., Armonk, NY, USA).

### 2.11. Transmission Electron Microscopy (TEM)

Cell preparation was performed according to a previously described protocol [11]. Cells grown on sterile Milinex plastic substrates (Agar Scientific, Essex, UK) were pre-fixed with 2.5% glutaraldehyde (Sigma-Aldrich, St. Louis, MO, USA) in a culture medium for 15 min, then fixed for 1 h with a 2.5% solution of glutaraldehyde in 0.1 M sodium cacodylate buffer (pH 7.3; Sigma-Aldrich, St. Louis, MO, USA), washed three times with the same buffer, and postfixed with a 1% solution of osmium tetroxide (Aurat) in the same buffer for 1 h at RT. After that, the cells were washed three times with distilled water, incubated in a 1% aqueous solution of uranyl acetate (Serva) for 16 h, and dehydrated in ethanol of increasing concentration (30–96%) and then in acetone. The dehydrated samples were embedded in the Agar-100 epoxy resin mixture (Fluka), which was polymerized at 60 °C for 48 h. Ultrathin sections (50–80 nm) were prepared with a diamond knife on a UCT7 ultramicrotome (Leica Microsystems, Wetzlar, Germany). The samples were analyzed under a JEM1400 transmission electron microscope (JEOL, Tokyo, Japan) at 80 kV at the Center for Microscopy of Biological Objects (Institute of Cytology and Genetics SB RAS, Novosibirsk, Russia, project 0324-2019-0042).

### 2.12. Scanning Electron Microscopy (SEM)

Cells grown on the chips were fixed in the growth medium containing 2.5% glutaraldehyde for 15 min and then transferred to a solution of 2.5% glutaraldehyde in 0.1 M cacodylate buffer for 1 h. Next, the chips successively underwent two washes in 0.1 M cacodylate buffer, were fixed in a 1% aqueous solution of osmium tetroxide, washed in double-distilled H_2_O, stained for 20 min in 1% uranyl acetate, and dehydrated by incubation in solutions of increasing concentration of ethanol (30%, 50%, 70%, and 100%) for 10 min each. The dehydrated samples were dried by the critical point protocol in a Critical Point Dryer (BAL-TEC, Balzers, Liechtenstein) and then examined at 10 kV under a scanning electron microscope (SU8220, Hitachi, Tokyo, Japan) before and after spraying a 1 nm chromium layer in an argon atmosphere (Coating Unit, Leica Microsystems, Wezlar, Germany).

### 2.13. Morphometric Analysis

Quantitative parameters of the spines of control (iso-control) and mutant neurons (iso 69Q group 1 and group 2) were evaluated on randomly selected sections obtained from three randomly selected areas of embedded samples. The operator was blinded to the assignment of sections to experimental groups. Spine length was measured on randomly selected dendrites, 50 spines per each group. The number of spines per dendrite length unit was calculated for 30 randomly chosen dendrites (per each group). For this, the number of spines and the length of each dendrite were counted, and the number of spines per 1 μm was calculated. The measurements were carried out in the ImageJ freeware (USA; ImageJ. Available online: https://imagej.nih.gov/ij/ (accessed on 5 November 2020)). The Student’s t test was used to determine statistical significance of differences between the average values.

## 3. Results

### 3.1. Generation of Human Cell Clones Harboring a CAG Expansion in the First Exon of the HTT Gene

**Figure 1:**
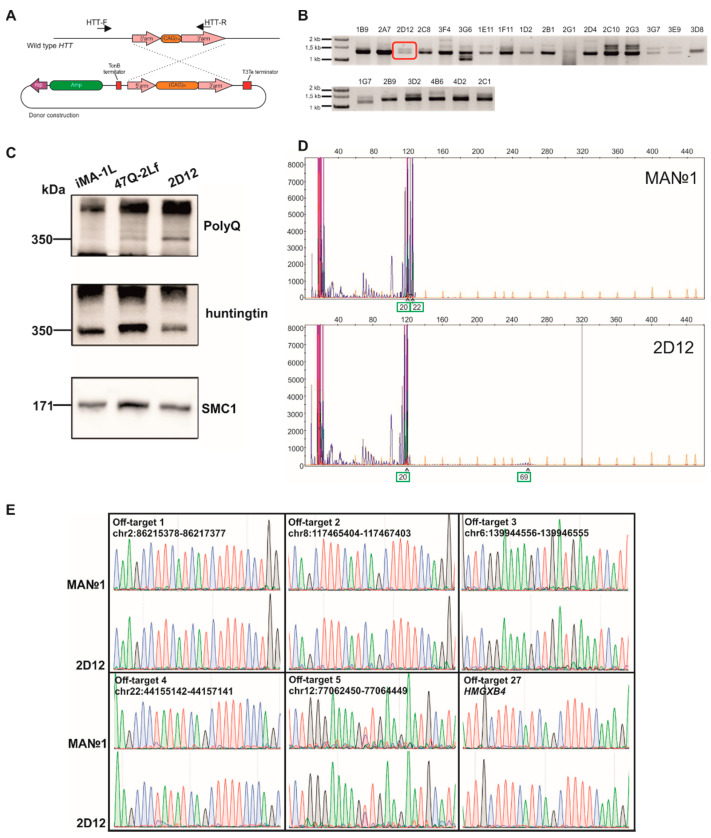
Generation of human embryonic fibroblast clones harboring the CAG expansion in the first exon of the *HTT* gene through CRISPR/Cas9-mediated homologous recombination: (**A**) The scheme of homologous recombination between wild-type *HTT* and the donor construct. HTT-F and HTT-R: primers for the detection of the insertion of the expanded CAG repeat tract into *HTT*; (**B**) PCR analysis of *HTT* allele lengths in the mutant cell clones. The clone selected for further study is highlighted by a red frame; (**C**) Western blot analysis of *HTT* expression in the mutant cells; (**D**) Fragment analysis of PCR products by capillary electrophoresis. Green squares and arrows indicate the number of CAG repeats in the *HTT* alleles; (**E**) Sequenograms of the off-target sites in the cell clones’ genomes. CRISPR/Cas9 (clustered regularly interspaced short palindromic repeats/CRISPR-associated protein 9).

To introduce an expanded CAG tract into *HTT*, we used a donor plasmid for homologous recombination at the *HTT* locus and a plasmid expressing elements of the CRISPR/Cas9 system, which were previously constructed in the Laboratory of Developmental Epigenetics (Institute of Cytology and Genetics, SB RAS) [9]. The donor vector contained long arms homologous to exon 1 of HTT: a 5′-arm (470 bp) and 3′-arm (1358 bp) flanking the 69-CAG repeat tract (Figure 1A). Human embryonic fibroblasts (named MA№1) were transfected via the polyethylenimine reagent with the donor and CRISPR/Cas9-expressing plasmids in an equimolar ratio. GFP-positive cells were sorted and plated at 1 cell/well in 96-well plates.

We analyzed 603 single-cell clones by PCR and found 23 clones with an expanded-CAG tract insertion (Figure 1B). The efficiency of homologous recombination at the *HTT* locus in the fibroblasts was ~4%, which is consistent with published data [12].

For further analysis, we chose clone 2D12 carrying a heterozygous insertion of the expanded CAG tract. Figure 1C shows the result of Western blot analysis with antibodies against the polyglutamine tract (polyQ) and the N-terminal huntingtin fragment (HTT); this analysis confirmed the expression of mutant huntingtin in clone 2D12. We designated a protein extract of original embryonic fibroblasts as an isogenic “healthy” control, and a protein extract of ICGi007-A cells (patient-specific iPSCs with 47 CAG repeats in exon 1 of *HTT*, 47Q) as a positive control [13].

To determine the number of CAG triplets in *HTT*, we performed capillary electrophoresis of fluorescently labeled PCR products. In Figure 1D, each peak corresponds to one CAG triplet. The reverse primer contained three CAG repeats in its sequence, and it is necessary to add 3 during the counting of the peaks. The first highest peak corresponds to the number of CAG repeats in one allele, and the second highest peak corresponds to that in the other allele. It was found that original embryonic fibroblasts contained 22 and 20 CAG repeats in the *HTT* alleles. Clone 2D12 contained 69 CAG repeats in the first allele and 20 repeats in the second allele (Figure 1D). The peaks corresponding to 69 CAG triplets are shorter than the peaks corresponding to the normal number of triplets owing to the decrease in amplification efficiency of the elongated CAG tract with an increasing triplet number [14].

### 3.2. The Off-Target Activity Assay of HTT-Targeted CRISPR/Cas9 in the Genome of Mutant Fibroblast Clones

We used the benchling.com Web service to search for off-target sites of the selected spacer in the human genome. This resource allowed us to find the most probable cleaved sequences in the genome of the research object (Table 3). As a result, the top five off-target sites in the human genome were selected for analysis. In addition, all the selected sites were located in noncoding regions of the genome. Furthermore, the 27th off-target site, which is located in the HMGXB4 protein-coding gene, was selected. Next, DNA sequences containing off-target sites (off-target sites 1 through 5 and off- target site 27) in the genome of wild-type embryonic fibroblasts and 2D12 cells were amplified, and then the PCR products were analyzed by Sanger sequencing. Figure 1E presents sequenograms of off-target sites in the genome of the cells. Under each off-target site, the target protospacer is presented; dots indicate nucleotide matches, and the letters substitutions. It should be noted that off- target site 1 differed by only two nucleotides from the target protospacer, with substitutions in the 5′ end of the sequence, thus making this site most susceptible to the cleavage by Cas9 [15,16]. In the case of cleavage of the off-target site and subsequent repair of the double-stranded DNA break by nonhomologous end joining, double peaks were likely to be detectable in the sequenogram. It was demonstrated that all the analyzed sequences remained intact (Figure 1E) in mutant cell clone 2D12, indicating that the activity of the selected *HTT*-targeted spacer was highly specific.

### 3.3. Generation and Characterization of iPSCs from the Mutant Fibroblasts

**Figure 2:**
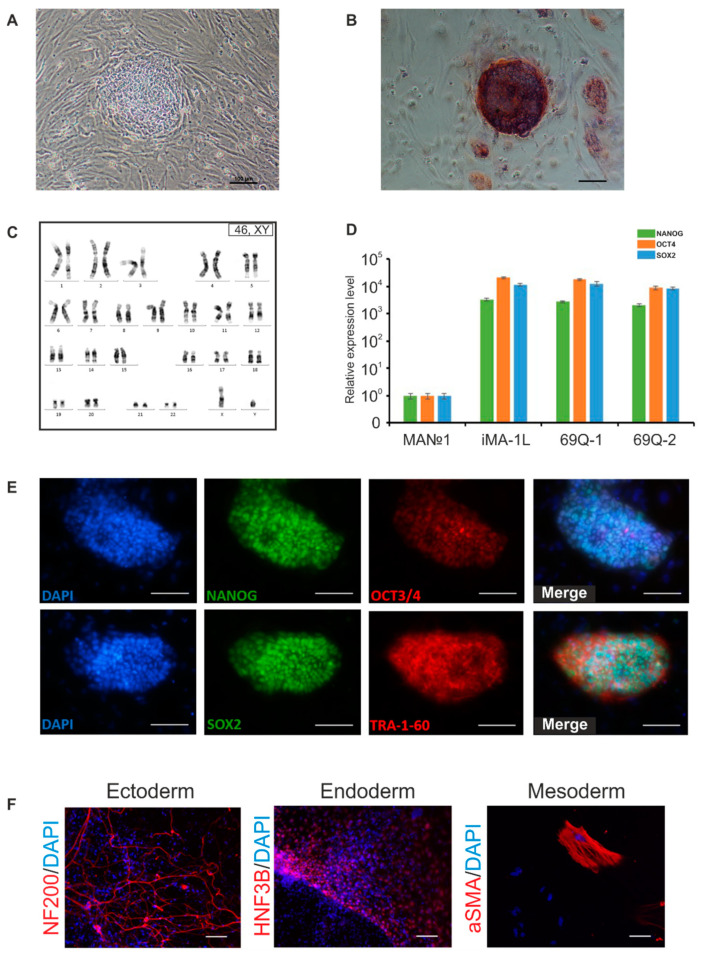
Detailed characterization of the induced pluripotent stem cells (iPSCs) derived from the mutant fibroblasts: (**A**) Morphology of a primary iPSC colony at 2 weeks after reprogramming initiation; (**B**) A colony of iPSCs expressing alkaline phosphatase; (**C**) The karyotype of iPSC clone 69Q-1; (**D**) Analysis of expression of pluripotency markers by qPCR (*n* = 3, values for biological replicates are presented as mean ± standard error of the mean); (**E**) Immunofluorescence staining of iPSC clone 69Q-1 with antibodies against transcription factors OCT3/4, NANOG, and SOX2 and surface antigen TRA-1-60; (**F**) Immunofluorescence staining confirming the expression of markers of three germ layers’ derivatives after spontaneous differentiation of clone 69Q-1. Scale bars: 100 µm.

For fibroblast reprogramming, we followed a previously described reprogramming protocol involving episomal vectors that do not integrate into the genome and cause transiently increased expression of pluripotency factors *OCT4*, *KLF4*, *L-MYC*, *SOX2*, and *LIN28* [17]. The episomes were delivered into cells by electroporation. Approximately on the 5–6th day of cultivation after the transfection, morphological changes of fibroblasts were noticed, the cells became rounded and formed small clusters. In the second week of cultivation, there were more compact groups of cells that formed cell colonies (Figure 2A). In the third week, large primary colonies of iPSCs with the most pronounced embryonic-stem-cell-like morphology were mechanically transferred onto a mouse embryonic fibroblast layer. The obtained iPSC lines proliferated rapidly, the cell colonies had flat single-layer morphology, and the cells expressed endogenous alkaline phosphatase (Figure 2B).

During the selection of recombinant fibroblast clones and subsequent reprogramming, the obtained cells underwent many divisions, which can lead to the accumulation of chromosomal abnormalities [18]. Therefore, the first step in the characterization of the cells was karyotyping, which detected no gain or loss of chromosomes and no major chromosomal aberrations (Figure 2C). By RT-qPCR, the expression of genes *NANOG*, *OCT3/4*, and *SOX2* was analyzed in the iPSC lines. It was found that pluripotency markers were expressed strongly in the iPSC lines but not expressed in fibroblasts (Figure 2D).

Immunofluorescence staining suggested that the iPSC lines expressed the main markers of pluripotent cells, such as surface antigen TRA-1-60 and transcription factors NANOG, OCT4, and SOX2 (Figure 2E). Spontaneous differentiation in vitro through formation of embryoid bodies revealed the expression of markers of three germ layers’ derivatives: the ectoderm (the main protein of the cytoskeleton of nerve cells NF200), the mesoderm (one of the main components of the contractile apparatus of smooth muscle cells: αSMA), and the endoderm (nuclear factor of hepatocytes 3B: HNF3B; Figure 2F).

### 3.4. Directed Differentiation of the iPSCs into MSNs

For this purpose, three iPSC lines were selected: 69Q, isogenic “healthy” control iMA-1L obtained by reprogramming of fibroblasts, and non-isogenic positive control iPSC 47Q obtained by reprogramming of patient-specific peripheral blood mononuclear cells carrying 47 CAG repeats in the first exon of the *HTT* gene [13].

**Figure 3:**
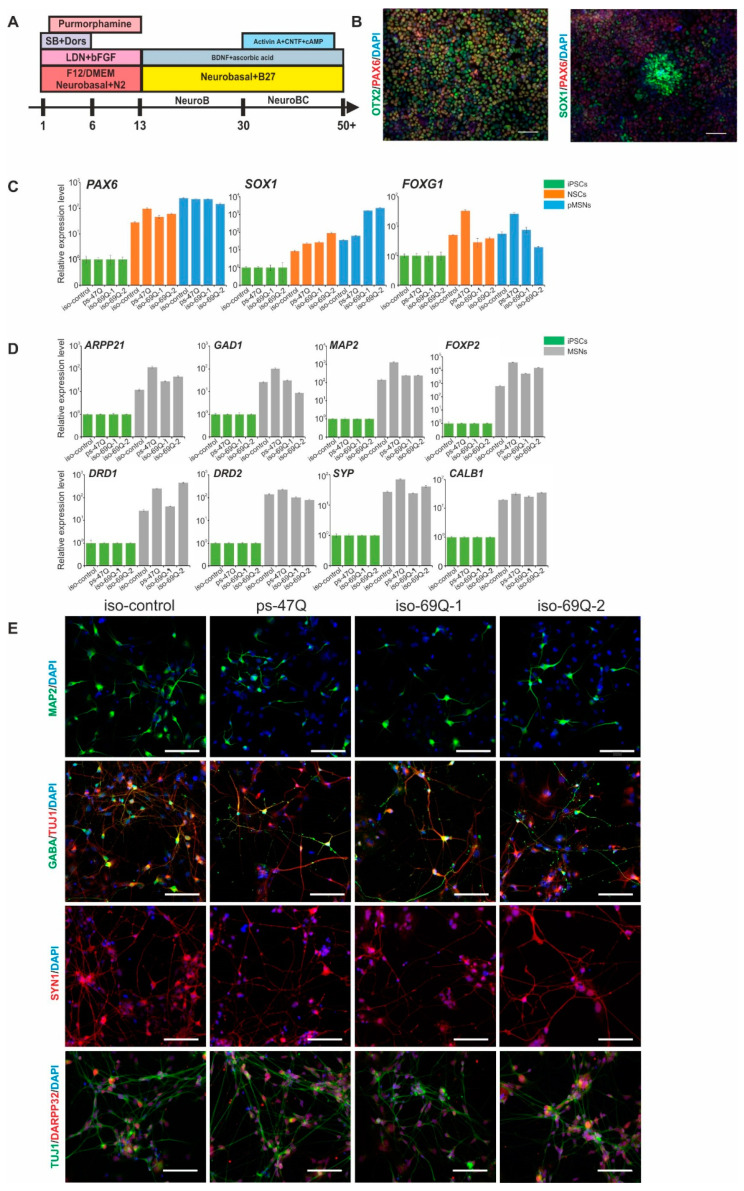
Characterization of the medium spiny neurons (MSNs) that developed from iPSCs: (**A**) The scheme of the iPSC differentiation into MSNs; (**B**) Immunofluorescence staining for markers of the early neuroectoderm; (**C**) Analysis of relative expression of early neuronal markers by qPCR; normalization to the level of expression in iPSCs (*n* = 3, values for biological replicates are presented as mean ± standard error of the mean); (**D**) Analysis of expression of MSN markers by qPCR; normalization to the level of expression in iPSCs (*n* = 3, values for biological replicates are given as mean ± standard error of the mean); (**E**) Immunofluorescence staining of neurons for markers of mature MSNs. Scale bars: 100 µm.

We followed the previously developed protocol of iPSC differentiation into MSNs based on 2D-cultivation and dual SMAD inhibition (Figure 3A) [10]. The first stage of differentiation leads to rapid neural induction and the formation of neuroectodermal cells after the addition of inhibitors SB431542 (TGFβ signaling inhibitor) and LDN193189 (BMP signaling inhibitor) to the culture medium. The cells expressed early markers of neuronal progenitor cells, e.g., PAX6 and SOX1 [19], as well as a specific forebrain marker, OTX2 [20] (Figure 3B).

The second stage of differentiation is the maturation and production of rapidly proliferating progenitor cells of MSNs (pMSNs). pMSNs expressed markers of neuronal central-nervous-system progenitor cells *PAX6* and *SOX1* and forebrain marker *FOXG1* on the 13th and 40th day of differentiation, as evidenced by qPCR (Figure 3C).

The last stage of differentiation is mature MSNs. During the terminal differentiation, the cells gradually stopped proliferating and formed long processes. Immunofluorescence staining showed the expression of such MSN markers as MAP2, GABA, DARPP32, and synapsin1 (SYN1) on day 20 of the terminal differentiation (Figure 3D). qPCR confirmed the strong expression of genes *MAP2*, *SYP*, *GAD1*, *ARPP21*, *CALB1*, *FOXP2*, *DRD1*, and *DRD2* (Figure 3E).

### 3.5. HD-Related Phenotypic Abnormalities in the Neural Derivatives Obtained from the Mutant Isogenic iPSCs

**Figure 4:**
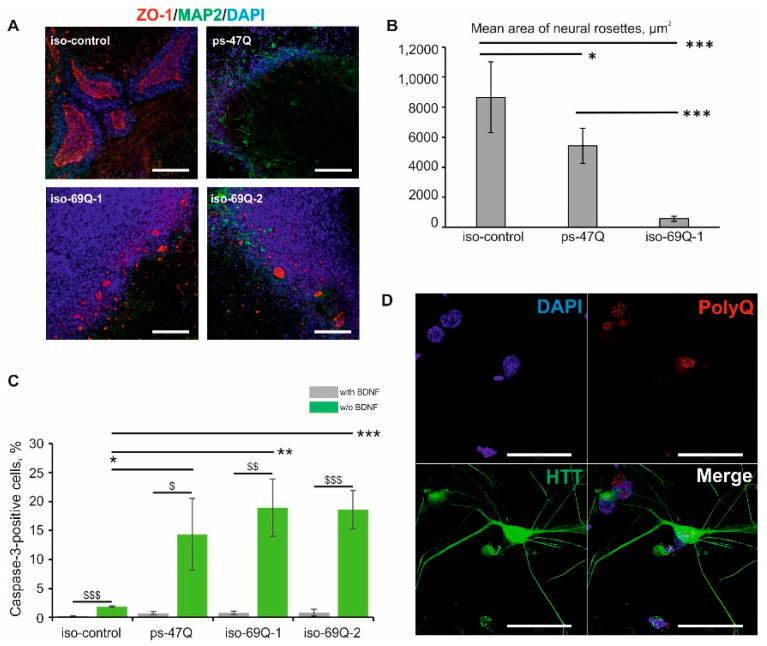
Phenotypic abnormalities in the neural derivatives of the mutant isogenic iPSCs: (**A**) The neural-rosette formation assay. Immunofluorescence staining for MAP2, a marker of neurons, and ZO-1, a marker of luminal rosettes. The results were reproduced in three independent experiments. Scale bar: 100 µm; (**B**) Mean area of neural rosettes; * *p* < 0.05, *** *p* < 0.001; (**C**) Increased sensitivity of mutant MSN (medium spiny neuron) progenitors to BDNF (brain-derived neurotrophic factor) withdrawal. Cell death was assessed by immunofluorescence staining with antibodies against activated caspase 3 after the removal of BDNF (*n* = 3, values for biological replicates are presented as mean ± standard error of the mean). * *p* < 0.05, ** *p* < 0.01, *** *p* < 0.001: a comparison between the cell lines after BDNF depletion; $ *p* < 0.05, $$ *p* < 0.01, $$$ *p* < 0.001: a comparison of one cell line before and after the BDNF depletion; (**D**) An immunofluorescence assay of neurons for the presence of mutant huntingtin aggregates. Scale bar: 50 µm.

In vitro research suggests that patient-specific iPSCs have an impaired ability to form neural rosettes [6,21]. Consistently with these studies, we observed marked impairment of neural-rosette formation in the patient-specific 47Q cell line compared with the iMA-1L control cell line (Figure 4A,B). In the isogenic 69Q cell line, the introduction of the HD mutation also negatively affected the neural-rosette formation (Figure 4A,B).

Increased cell death of the neurons and neuronal stem cells that developed from HD iPSCs after the growth factor withdrawal has been reported previously [4,6]. In line with these studies, we observed increased cell death following the growth factor withdrawal in the pMSNs that developed from iPSC lines 47Q and 69Q as compared to “healthy” control line iMA-1L, as revealed by caspase 3 immunofluorescence staining (Figure 4C). In contrast, the mutant progenitors did not show significant differences in the number of activated-caspase-3-positive cells when compared with each other. The consistent vulnerability of HD pMSNs to the growth factor withdrawal confirms the contribution of trophic support deficits to the pathogenesis of HD.

Nuclear and cytoplasmic inclusions of mutant huntingtin in neurons are the hallmark of HD [22]. Nevertheless, we did not find huntingtin- or polyQ-positive aggregates after immunofluorescence staining with antibodies against huntingtin (N terminus) and against polyQ (the antibody binds to proteins containing >39 glutamine residues) in MSN clones 69Q and 47Q, in agreement with studies on cultured human cells (Figure 4D) [4].

### 3.6. Ultrastructural Characteristics of the Isogenic HD Neurons

**Figure 5:**
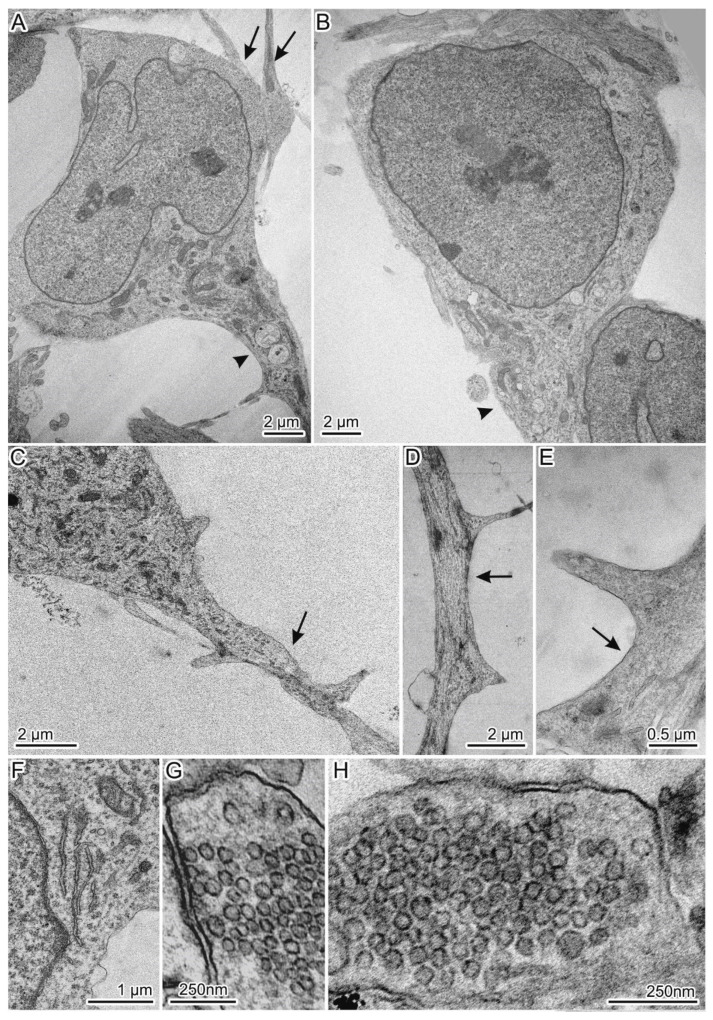
General view of spiny neurons with normal *HTT*; (**A**,**B**) Triangular neurons with large nuclei and an axon and dendrites extending from the first of them; (**C**–**E**) Spines on dendrites can be oriented in one or different directions; (**F**) A Nissl body; (**G**,**H**) Synapses contain synaptic vesicles of similar sizes. Axons and dendrites are indicated by arrowheads and arrows correspondingly.

The isogenic MSNs were investigated by electron microscopy. The neurons derived from the “healthy” control line had typical morphology of MSNs (Figure 5) [10]. The cells were triangular or rounded, with a large nucleus, axon, and spines on the dendrites (Figure 5A–C). The spines were distributed on the dendrites as follows: one or two at a distance of 6 µm or more (Figure 5C–E). Such characteristic structures as neurofilaments and Nissl bodies were present too (Figure 5F). Furthermore, in the culture of the control neurons, typical synapses were found with presynaptic club-shaped endings containing a large number of synaptic vesicles of approximately the same size (Figure 5G,H). “Healthy” neurons contained mitochondria with a predominantly elongated shape, transversely located cristae, and the matrix of medium density; they were distributed all over the cytoplasm. Abnormalities in the structure of mitochondrial cristae and matrix density were rarely found together with moderately pronounced mitophagy. The cytoplasm contained the endoplasmic reticulum (ER) presumably of the smooth type, the Golgi complex, lysosomes, autolysosomes, and a small number of vacuoles of different sizes (Figure 5).

**Figure 6:**
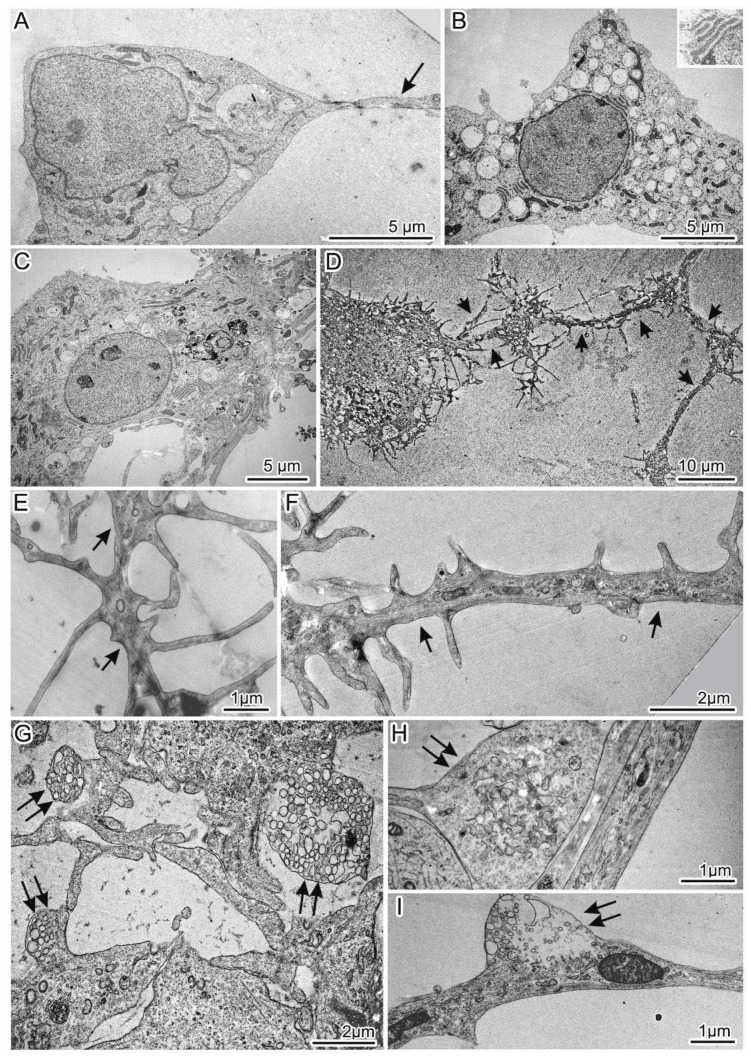
Morphological characteristics of the cultured 69Q spiny neurons: (**A**) Shape of group 1 mutant neurons is similar to that of control cells; (**B**) The first subtype of neurons has a large number of light vacuoles filling the cytoplasm. Inset: A Nissl body at high magnification; (**C**) The second subtype of neurons contains large aggregates of autophagosomes and autolysosomes in the cytoplasm; (**D**) General view of a neuron from group 2 with abnormal morphology and containing multiple atypical dendrites and spines; (**E**,**F**) Elongation of spines and a high frequency of their location on the dendrite of a neuron from group 2; (**G**–**I**) Defective synapses with abnormal size, organization, and distribution of synaptic vesicles. Dendrites and defective synapses are indicated by black arrows and double arrows correspondingly.

**Figure 7:**
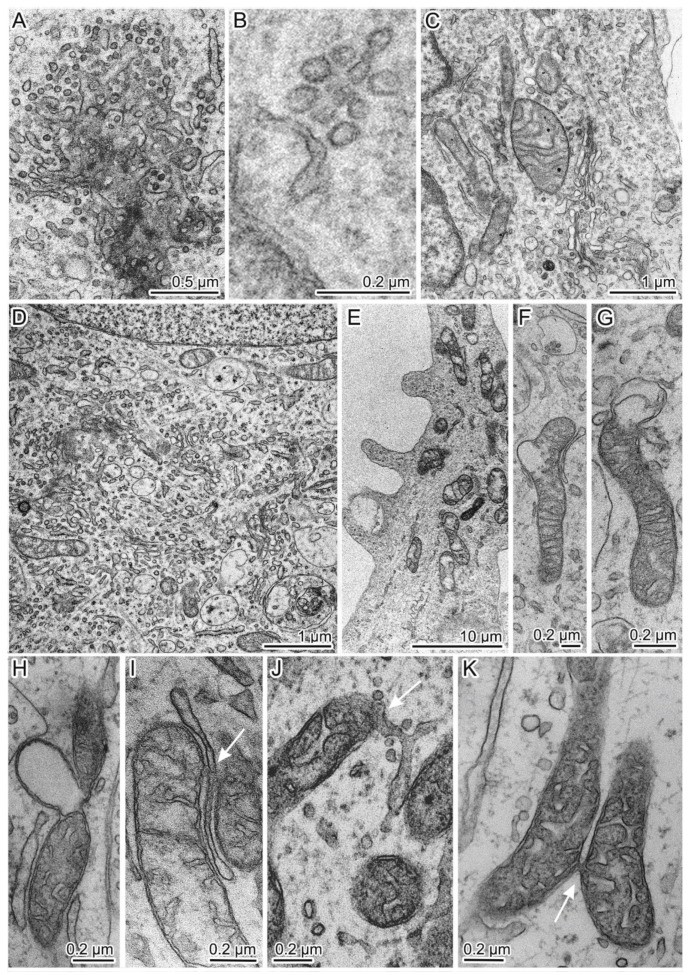
Pathological changes in the organization of cytoplasmic organelles in the mutant neurons of group 1: (**A**) Dense accumulation of small vesicles near smooth membranes of the ER and Golgi complex; (**B**) Accumulation of small vesicles near the smooth-ER membranes in a “healthy” control neuron; (**C**) The Golgi complex in a control cell; (**D**) High density of small vesicles and actively functioning Golgi cisterns in the cytoplasm of a 69Q neuron; (**E**–**H**) Defective mitochondria with a sparse matrix and impaired envelope integrity; (**I**,**J**) A contact of defective mitochondria with smooth- ER membranes; (**K**) A contact between defective mitochondria. Contacts are indicated by white arrows.

**Figure 8:**
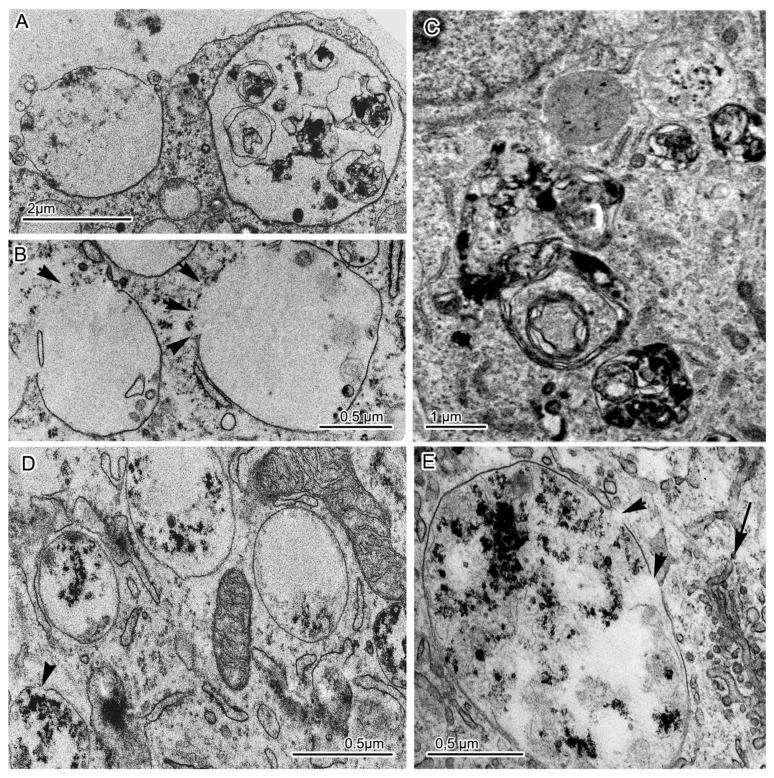
Disruption of the integrity of membranes of light vacuoles and autolysosomes in the cytoplasm of the mutant neurons: (**A**) The release of the contents of a light vacuole into intercellular space by exocytosis; (**B**) Breaks in the envelope of light vacuoles (indicated by black arrowheads), leading to the release of their contents into the cytoplasm; (**C**) Aggregates of autophagosomes and autolysosomes in the cytoplasm of a subtype 2 neuron; (**D**,**E**) Disruption of the integrity of the autolysosome membrane (indicated by black arrowheads). The black arrow points to the accumulation of small vesicles near smooth-ER membranes.

**Figure 9:**
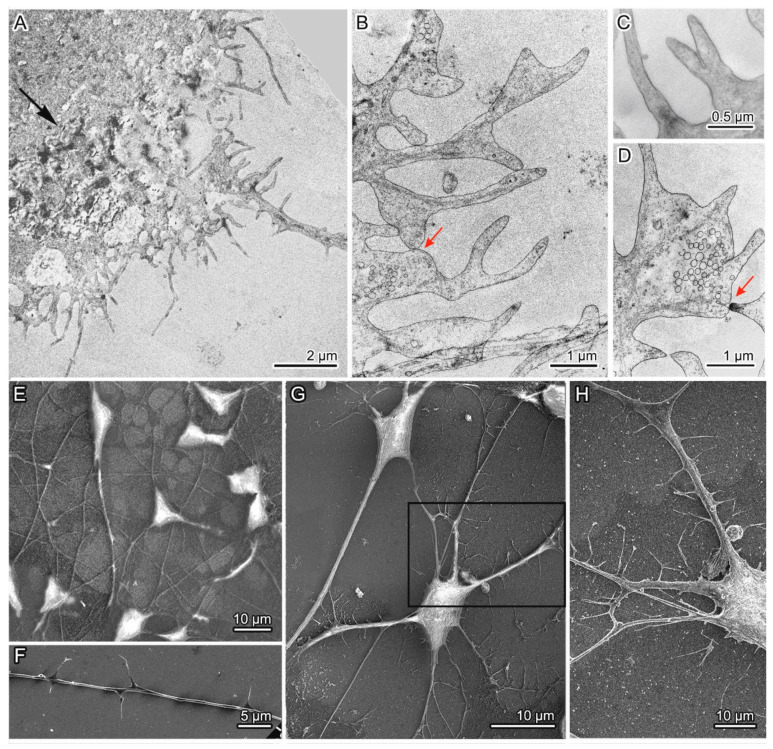
Abnormal organization of some neurons among group 2 mutant neurons: (**A**) A part of an atypical-morphology neuron containing a dense aggregate in the cytoplasm (indicated by a black arrow) and a large number of short and long dendrites with spines randomly located on them; (**B**,**C**) Spines with a defective shape: bifurcation or adhesion of closely spaced structures; (**D**) A contact of defective spines and a synapse of abnormal organization; (**E**,**F**) Neurons and a dendrite with spines in the control culture; (**G**) A mutant neuron from group 1 (left) and an atypical neuron from group 2 (right) with dendrites and spines; (**H**) An enlarged part of an atypical neuron showing a large number of spines on the dendrite. The same fragment is highlighted in the frame in the panel G. Images in panels A–D were obtained by TEM, and in panels E–H via SEM. Contacts of spines and synaptic terminals are indicated by red arrows.

We subdivided the population of mutant 69Q neurons into two types by morphological features. Group 1 mutant neurons included the main cell type and two subtypes (Figure 6). All these cells had the cytoplasm filled with organelles with greater density as compared to control neurons and were characterized by numerous defects in their ultrastructure (Figure 7 and Figure 8). The first cell type was the most common and matched the typical neuronal shape (Figure 6A); the cells of subtype 1 were characterized by the presence of many large light vacuoles in the cytoplasm (Figure 6B and Figure 8A,B), whereas the cells of subtype 2 contained congestions of large autophagosomes and autolysosomes (Figure 6C or Figure 8C–E). The number of spines on the dendrites of group 1 neurons was comparable to or lower than that in the control (Figure S1). Group 2 mutant neurons included unusual cells characterized by dense cytoplasm and abnormal (mutation-induced) hypertrophic development of dendrites and impaired spines (Figure 6D or Figure 9 or Figure S2). The length of many spines and the frequency of their distribution on dendrites were substantially greater (Figure 6E,F or Figure S1). Some thin scions formed by the cell membrane were also observed (Figure 6D or Figure 9A). Abnormal synapses contained synaptic vesicles that were different in size or formed defectively and featured an unusual distribution (Figure 6G–I).

Abnormal (mutation-induced) features of the neurons of the main cell type included large clusters of small vesicles, which often localized and assembled in contact with smooth-ER and Golgi complex membranes (Figure 7A). Similar small clusters were observed in the cytoplasm of control neurons (Figure 7B). Activity of the Golgi complex was substantially higher in the mutant cells (Figure 7D) than in the control cells (Figure 7C). Low density of the mitochondrial matrix, deformed cristae, and impaired integrity of their envelope were often seen in the mutant cells’ dendrites (Figure 7E) and in the soma cytoplasm (Figure 7F,G). Tight contacts between defective mitochondria and ER membranes (Figure 7I,J) as well as intermitochondrial interactions were identified too (Figure 7K). Excretion of large vacuoles from the cytoplasm via exocytosis into extracellular space was noted in subtype 1 neurons (Figure 8A). Numerous fragmented disturbances and massive disruptions of vacuole membrane integrity were identified in the cytoplasm of these cells (Figure 8B). The subtype 2 neurons containing large aggregates of autophagosomes and autolysosomes (Figure 8C) featured similar defects of autolysosome membranes (Figure 8D,E). Neurons from group 2 showed abnormal organization (Figure 9A–D,G,H), which completely differed from the organization of control neurons (Figure 9E,F). They contained spines of abnormal morphology, e.g., with bifurcation (Figure 9B) or adhesion (Figure 9C). Sometimes, they were in contact with defectively organized synapses (Figure 9D or Figure S2). Identical defects in the structural organization of neurons from group 2 were registered by TEM (Figure 6D–F) and SEM (Figure 9E–H).

## 4. Discussion

The CRISPR/Cas9 system has made the creation of isogenic models of hereditary human diseases more efficient. In this work, CRISPR/Cas9-mediated homologous recombination was implemented to introduce an expanded CAG tract into the first exon of the *HTT* gene of human cells for HD modeling. We show that isogenic iPSCs with the introduced mutation in *HTT* retain pluripotency and a normal karyotype and can be differentiated into the specific neurons that are mostly affected by HD. We additionally demonstrate that several phenotypic abnormalities of HD iPSC-derived neural cells, including impaired neural-rosette formation and increased susceptibility to growth factor withdrawal, are reproduced in the isogenic mutant cells. Moreover, by electron microscopy, we revealed many apparent ultrastructural defects when comparing an isogenic “healthy” control line with the MSNs that developed from patient-specific iPSCs and iPSCs carrying the introduced HD mutation. These data indicate that these differences are likely related to HD-specific effects, and the generated isogenic cells mimic the pathological phenotype.

Creation of a panel of isogenic HD iPSCs by introduction of the HD mutation into normal cells has been performed previously by TALEN and CRISPR/Cas9-assisted methods [5,8]. On the other hand, in those studies, donor vectors containing a selection cassette were introduced into the genomic locus of *HTT* after the targeting, and additional cassette removal manipulations may have undesirable consequences. Our selection cassette-free approach is important for obtaining an optimal disease phenotype.

Off-target activity evaluation is a key task during genome editing by the CRISPR/Cas9 system. According to published data, the CRISPR/Cas9 system can introduce double-strand breaks at off- target genomic sites that differ from the target sequence by several nucleotides [23,24]. Here, we verified the genomic regions that carry the highest risk of inappropriate modification by CRISPR/Cas9 using Sanger sequencing. We demonstrated that all these sequences remained intact in the genome-edited cells. This finding suggests that the activity of the selected CRISPR/Cas9 spacer is specific to the *HTT* locus.

Transcription factors OCT3/4, SOX2, and NANOG play a crucial role in the extensive regulatory system maintaining self-renewal and pluripotency of cells, and increased expression of these proteins is an important characteristic of iPSCs. Another crucial property of iPSCs is pluripotency, i.e., the ability to differentiate into the cell types of three germ layers: the ectoderm, endoderm, and mesoderm. In our study, fibroblast clones with the introduced mutation were reprogrammed, and various methods were applied for confirming their pluripotent state.

The main neuropathological feature of HD is selective death of striatal MSNs. In this study, using our previously published protocol [10], we derived neurons expressing the basic neuronal markers such as MAP2 and SYN1 as well as MSN-specific markers such as DARPP32, ARPP21, CALB1, FOXP2, DRD1, and DRD2 and neurotransmitter GABA [8].

Many studies indicate that huntingtin is essential for normal brain development [25,26]. Additionally, patient-specific iPSCs manifest impaired neural-rosette formation [6,21]. In line with these studies, we observed a marked impairment of neural-rosette formation in both edited and patient-specific iPSCs as compared to control iPSCs. This disturbance is likely a consequence of the mutation and is not related to differences in the genetic background.

Caspase 3 is a key mediator of apoptosis of neurons in the pathogenesis of many neurodegenerative diseases [27]. In patients with HD, caspase 3 activity is increased in lymphoblasts [28]. Moreover, this feature is observed under stress: e.g., incubation of lymphoblasts with cyanide or culturing of neural stem cells in a medium without growth factors [4,6,29]. Besides, one of the indicators of the pathological phenotype of HD-affected cells is differential sensitivity of mutant cells to the withdrawal of growth factors.

Indeed, we noticed a significant increase in the number of activated-caspase-3-positive cells in the experiment with the mutant progenitors of MSNs 47Q and 69Q after the deprivation of BDNF as compared to the control cells. This result reproduced the well-characterized phenotype seen in various HD models, and the mutation introduced into *HTT* contributed to the manifestation of this phenotype. The increased sensitivity of mutant cells to the removal of growth factors confirms the contribution of trophic reinforcement deficiency (in particular, impaired BDNF signaling in brain cells) to HD pathogenesis. BDNF is not expressed in cells of the lateral ganglionic eminence (progenitors of striatal cells) and in the striatum. This trophic factor is anterogradely transported to the striatum from cells of the cerebral cortex, substantia nigra pars compacta, and thalamus. On the other hand, mutant huntingtin disrupts the expression and transport of BDNF and thereby reduces the viability of progenitor cells and causes neuronal dysfunction and striatum atrophy [30]. Consequently, the significant effect of BDNF absence in the culture medium on the viability of the control progenitors (an increase in the number of caspase-3-positive cells from an average of 0.28% to 1.87%) can be explained by narrower specialization in the striatal direction, and probably some proportion of the cells does not express BDNF. For the mutant progenitors, the significant change in cell viability (an increase in the number of caspase-3-positive cells from 0.8% to 17.3% on average) is due to the aforementioned negative effect of mutant huntingtin on BDNF signaling.

HTT-positive protein aggregates in MSNs were not detected here, consistent with the data from many research groups who studied neurons derived from patient-specific iPSCs [4,21,31,32]. These neurons did not show obvious neuropathological changes, and cell survival decreased only after exposure to agents causing excitotoxic or oxidative stress. Additionally, mutant-huntingtin aggregates emerged only after prolonged cultivation in vitro and in vivo (more than 6 months) or exposure to proteasome inhibitors. Thus, the late manifestation of HD makes patient age an important factor, which should be properly reflected in the experimental model. One of the possible reasons for the absence of aggregates in the neurons derived from iPSCs may be the reprogramming process, when the cells lose their “age-related” epigenetic modifications. Recent research suggests that the age-dependent HD phenotype is better reproduced by striatal MSNs obtained by direct transdifferentiation of patient-specific fibroblasts [33,34].

With the help of TEM and SEM, we characterized a wide spectrum of abnormalities in the ultrastructure of iPSC-derived striatal MSNs with 69 CAG triplets in *HTT*. The neurons were characterized by higher density of lysosome-like small vesicles in the cytoplasm as compared to the control, aberrant structural organization of mitochondria, mitophagy, and high activity of the smooth ER and Golgi complex. Alterations in the lysosomal system included accumulation of large autophagic vacuoles, autolysosomes, and autophagolysosomes in some cells as well as disruption of their membrane integrity. Defects in the structural organization of synapses were noted, and a new pathological type of neurons characterized by hypertrophic development of dendrites with high density of abnormally organized spines was revealed featuring a large number of spines on the dendrites in contact with other neurons.

Striatal MSNs have multipolar morphology: they consist of a soma, axon, and many dendrites. A characteristic feature of MSNs is the presence of a large number of spines on dendrites; for this reason, the total surface for receiving signals from other neurons is larger.

In vitro, neurons may form synapses, i.e., structures representing a contact of the axon terminal of one neuron with the dendrite (or spine) of another neuron [35]. A neural network is formed by synapses when a signal from one neuron is transmitted to another one and represents one piece of evidence for functional activity of cultured neurons. Defective synapses with different sizes of vesicles and anomalous organization seen in the mutant cultured neurons are most likely dysfunctional. It is believed that at early stages of the development of some diseases, there is an impairment of synaptic transmission [36]. Perhaps, this is due to axonal-transport dysfunction, which has been observed in HD. This phenomenon has been previously detected in ultrastructural studies, when researchers have documented variations in the number of synaptic vesicles and abnormal profiles of membrane organelles in the axons of the affected neurons [37,38]. Thus, the deficit of axonal transport may represent a serious neuropathological event underlying the selective death of striatal MSNs in HD.

Mitochondria are the main producers of energy in neurons and are involved in the regulation of Ca^2+^ homeostasis, cell stress, and many other processes [39], whereas changes in mitophagy and in the functioning of mitochondria cause disturbances in energy metabolism and increase oxidative stress, which ultimately results in dysfunction of neurons and their death in HD. The presence of mitochondria defective in the matrix and cristae in 69Q MSNs is probably associated with aberrations in mitochondrial division and fusion processes or in mitophagy, thereby resulting in the accumulation of damaged mitochondria and subsequently oxidative stress and their dysfunction [40,41]. Normally, the contacts between the ER and mitochondria are few and associated with the transport of ions, lipids, and calcium ions into the mitochondria [42]. The dysfunction of these contacts leads to the development of neurodegenerative diseases [43]. Similar abnormally extended contacts have been previously documented in our study on HEK293 cell ultrastructure with an elongated CAG repeat tract [11]. We propose that the increase in the number of mitochondrial contacts with ER cisterns in the mutant cells may contribute to increased concentration of calcium ions in mitochondria and to a release of cytochrome *c* from mitochondria, which in turn leads to apoptosis [44].

Lysosomes, autolysosomes, and autophagosomes are components of the lytic system, which maintains a balance between biogenesis and degradation of macromolecules and includes autophagy, by which the cell gets rid of “aging” organelles [45]. Because of the longer “lifespan” and increased activity, neurons are especially sensitive to the dysfunction of these organelles. The dependence of neurons on optimal functioning of lysosomal machinery has been confirmed as a correlation between lysosomal dysfunction and a wide range of neurological diseases [46,47].

Abnormal activation of the autophagic/lysosomal system is a common feature of various neurodegenerative disorders and diseases associated with changes in protein conformations. In addition to HD, an increased number of autophagolysosomes in the cell has been described in Alzheimer’s disease, Parkinson’s disease, and prion diseases [48]. Nonetheless, the reasons for the increased autophagic compartments differ among diseases and range from increased regulation of autophagosome formation to impaired autophagosome/lysosome fusion or ineffective degradation of cytoplasmic components after delivery to lysosomes. Currently, there are no reports of disrupted integrity of the autophagolysosome membrane in HD, and we have observed this phenomenon previously, in our study on morphology of mutant HEK293 cells carrying an expanded CAG tract in the *HTT* gene [11]. Permeabilization of autophagolysosome membranes in 69Q neurons may be a consequence of oxidative stress and an increase in the concentration of reactive oxygen species due to the mitochondrial dysfunction. The bottling of autophagolysosome contents will most likely induce cell death. Our data on the activation of the lysosomal system in the mutant neurons are consistent with the results in our previous publications [32] and with implications of studies on mutant neurons derived from iPSCs obtained from HD patients [48,49].

In the present study, morphological defects of MSN synapses, dendrites, and spines were observed at intermediate and late stages of HD mutant neuron cultures. These alterations were mostly manifested in the atypical cells of group 2, first identified in culture and characterized by considerable proliferative changes. The latter included severe bending of distal dendrite segments, branching of short segments, and increased spine density along dendrite length as well as a large variation in spine size. It should be noted that in the neurons of group 1, the number of spines on dendrites was comparable to or lower than that in the control. The morphological changes observed in the neurons carrying the HD mutation are in agreement with the literature data [50,51,52,53].

Spines on dendrites represent a filopodium-like protrusion and mushroom-shaped morphology, and the actin cytoskeleton plays a key part in their formation [54]. A decrease in spine density has been demonstrated in animal models of HD [55]. Actinin-2 is an actin filament cross-linker, localizes to dendritic spines, is enriched within the post-synaptic density, and is implicated in actin organization. At present, the biological role of actinin-2 in the spine is not clear. Overexpression of α- actinin-2 increases the length and density of dendritic protrusions in cultured hippocampal neurons, implying a function in the determination of spine morphology [56]. A loss of α-actinin-2 strongly increases spine density [57]. It is possible that the presence of abnormal mutant neurons in 69Q MSN culture that have unusual organization of dendrites and high spine density is probably related to α-actinin-2 upregulation.

## 5. Conclusions

In this study, we generated a novel isogenic HD model based on an iPSC line carrying 69/22 CAG repeats in the first exon of the *HTT* gene with a control isogenic iPSC line without this mutation. The expanded CAG tract was introduced via CRISPR/Cas9-mediated homologous recombination. The pluripotent status of the obtained cell lines was confirmed by a number of standard tests. In addition, we evaluated CRISPR/Cas9 off-target activity. The neurons derived from these iPSCs express the main markers of mature striatal neurons and possess the pathological phenotype of HD. Using TEM and SEM, we investigated pathological changes in the mutant neurons. Our study revealed typical-for-HD morphological defects in structural organization and interactions of mitochondria and the smooth ER as well as activation of the autophagic/lysosomal system. A type of atypical mutant neuron with well-pronounced development of defective dendrites and high spine density was identified for the first time. Our results mean that the obtained isogenic cell system can serve as a valuable model for the investigation into various parameters of HD and for screening of potential drugs without worries about background-related variability. In addition, the finding that these neurons have an embryonic phenotype makes it possible to study early pathological changes in HD. This research in turn can help to design a preventive modality against the disease, thereby not only delaying but also possibly preventing the onset of HD symptoms.

## Figures and Tables

**Table 1:** Oligonucleotides used for RT-qPCR.

Gene	5′→3′
*ACTB*	AGGCACCAGGGCGTGAT/GATAGCACAGCCTGGATAGCA
*NANOG*	CAGCCCCGATTCTTCCACCAGTCCC/CGGAAGATTCCCAGTCGGGTTCACC
*OCT3/4*	CTTCTGCTTCAGGAGCTTGG/GAAGGAGAAGCTGGAGCAAA
*SOX2*	GCTTAGCCTCGTCGATGAAC/AACCCCAAGATGCACAACTC
*HPRT*	GACTTTGCTTTCCTTGGTCAGG/AGTCTGGCTTATATCCAACACTTCG
*SOX1*	ATTATTTTGCCCGTTTTCCC/TCAAGGAAACACAATCGCTG
*PAX6*	TCCGTTGGAACTGATGGAGT/GTTGGTATCCGGGGACTTC
*MAP2*	TTCGTTGTGTCGTGTTCTCA/AACCGAGGAAGCATTGATTG
*SYP*	CAATGCCTGCCTGAACAAAG/GGGTCCTAAACTGTCCTCTCTA
*ARPP21*	CTGGATGAAGAGGAGAAACTGG/CCTGCTCCTGACTTGGATTT
*GAD1*	AAACCGTGCAATTCCTCCTG/GCAACTGGTGTGGGTGATGA
*CTIP2*	AGTGGCCGCGTGTATATTG/GCCCAGGCATTCTCGATTATTA
*DRD1*	CAACCTGAACTCGCAGATGAA/CAGAGTCTCACCGTACCTTAGT
*DRD2*	CACTCCTCTTCGGACTCAATAAC/GACAATGAAGGGCACGTAGAA
*CALB1*	CCGAACGGATCTTGCTCTTAT/ACTCCCTTATAGTGCACAGTTATT
*FOXP2*	CCAAAGCATCACCACCAATAAC/CTGTCTCGTCTTGCACTTAGAA

RT-qPCR—quantitative reverse transcription PCR.

**Table 2:** Antibodies used for immunocytochemistry.

Antibody	Company, Cat number	Dilution
**Primary**		
Rabbit IgG anti-GABA	Sigma, ABN131	1:500
Mouse IgM anti-PAX6	Santa Cruz Biotechnology, sc-81,649	1:50
Goat IgG anti-SOX1	R&D Systems, AF3369	1:200
Goat IgG anti-OTX2	R&D Systems, AF1979	1.400
Mouse IgG2a anti-TuJ1	Covance, 801201	1:1000
Rabbit IgG anti-NF200	Sigma, N4142	1:1000
Rabbit IgG anti-DARPP32	Abcam, ab40801	1:50
Chicken IgG anti-MAP2	Abcam, ab5392	1:1000
Rabbit IgG anti-SOX2	Cell Signaling Technology, 3579	1:500
Mouse IgM anti-TRA-1-60	Abcam, ab16288	1:200
Mouse IgG anti-NANOG	Abcam, ab62734	1:200
Mouse IgG anti-OCT4	BD Transduction Lab, 611,202	1:50
Mouse IgG anti-αSMA	DAKO, M0851	1:100
Rabbit IgG anti-HNF3B	Millipore, 07-633	1:200
Rabbit IgG anti-HTT	Sigma, H7540	1:100
Mouse IgG anti-PolyQ	Millipore, MAB1574	1:400
Rabbit IgG anti-Cleaved caspase-3	Cell Signaling Technology, 9661	1:400
Mouse IgG1 anti-ZO-1	Invitrogen, 33-9100	1:50
Rabbit IgG anti-Synapsin I	Millipore, 574,777	1:200
**Secondary**		
Alexa Fluor 488 goat anti rabbit IgG (H+L)	Thermo Fisher Scientific, A11,078	1:400
Alexa Fluor 568 goat anti rabbit IgG (H+L)	Thermo Fisher Scientific, A-11,011	1:400
Alexa Fluor 488 goat anti mouse IgG (H+L)	Thermo Fisher Scientific, A28,175	1:400
Alexa Fluor 568 goat anti mouse IgG (H+L)	Thermo Fisher Scientific, A11,031	1:400
Alexa Fluor 568 goat anti mouse IgG2a	Thermo Fisher Scientific, A-21,134	1:400
Alexa Fluor 488 goat anti mouse IgG1	Thermo Fisher Scientific, A-21,121	1:400
Alexa Fluor 568 goat anti mouse IgM	Thermo Fisher Scientific, A21,043	1:400
Alexa Fluor 488 rabbit anti goat IgG (H+L)	Thermo Fisher Scientific, A27,012	1:400
Alexa Fluor 488 goat anti chicken IgY H&L	Abcam, ab150,169	1:400

**Table 3:** Top 27 off-target sites in the human genome for the spacer of the HTT-targeted CRISPR/Cas9 system.

Sequence	PAM	Score	Chromo-Some	Gene	DNA Strand	Substitutions
AGGCCTTCATCAGCTTTTCC	AGG	100	4	*HTT*	-	0
AAGCCTTCATCATCTTTTCC	TGG	3.60441176	2		-	2
CAGCTTTCATCAGCTTTTCC	AGG	2.50329381	8		+	4
ATGATTTCATCAGCTTTTCC	GAG	2.4265645	6		-	3
AGCCCTATATCAGCTTTTCC	CAG	1.74086014	22		+	4
ATGCATTCTTCAGCTTTTCC	CAG	1.68981077	12		+	3
GGGCCCTCAGCAGCTTTTCC	TGG	1.65679108	5		+	3
AGCCTTTCATGAGCTTTTCC	CAG	1.56823077	2		-	3
CAGCCTTCATCAGCTTCTCC	AAG	1.55307856	22		-	3
CAGCTTTAATCAGCTTTTCC	TAG	1.50316456	4		-	4
TGGTCTTAAGCAGCTTTTCC	TAG	1.47132848	5		+	4
TTTCCTTCAACAGCTTTTCC	TGG	1.41271949	11		-	4
AGTCCTCCATCAGCTTTTCT	AAG	1.19365756	8		+	3
GAGCCTGGATCAGCTTTTCC	TGG	1.04428648	14		-	4
TGGGCTGAATCAGCTTTTCC	TGG	1.02666139	6		+	4
TGGCTTCCAGCAGCTTTTCC	AAG	0.98720955	7		-	4
ATGGCTGCAGCAGCTTTTCC	AAG	0.97011502	X		-	4
AAGAATCCATCAGCTTTTCC	AAG	0.96173419	2		+	4
AATTCTACATCAGCTTTTCC	TAG	0.94826991	9		+	4
AAATCTCCATCAGCTTTTCC	GAG	0.94826991	6		-	4
TGCCCTTGGTCAGCTTTTCC	TGG	0.94546867	20		-	4
AGGGCCTCATGAGCTTTTCC	AAG	0.92863537	9		+	3
AGGGCTGTAGCAGCTTTTCC	CAG	0.90727356	16		+	4
AGGGCTGTAGCAGCTTTTCC	CAG	0.90727356	16		+	4
TAGCATTCATCAGCTTTTCA	AGG	0.90034091	5		+	4
TAGCTTTCATCAGCTTTTCA	AAG	0.90034091	X		-	4
TAGCTTTCATCAGCTTTTCA	AAG	0.90034091	11		+	4
TTGCTTTCATCAGCTTTTCA	AAG	0.90034091	22	*HMGXB4*	+	4

PAM: protospacer-adjacent motif. The target protospacer is highlighted in green, and the studied off-target sites in orange.

## Supplementary Materials

The following are available online at https://www.mdpi.com/2075-4426/10/4/215/s1, Figure S1: Quantifications of spine length and density in mutant neurons and isogenic healthy control. *** p < 0.001; Figure S2: Atypical spines of mutant neurons contacting with synaptic terminals (synaptic terminals are indicated by double black arrows).
