# Supplementary figures and images for: A Human Induced Pluripotent Stem Cell-Derived Isogenic Model of Huntington’s Disease Based on Neuronal Cells Has Several Relevant Phenotypic Abnormalities
Source: J Pers Med. 2020 Nov 9;10(4):215. doi: 10.3390/jpm10040215 (PMC7712151; doi:10.3390/jpm10040215)

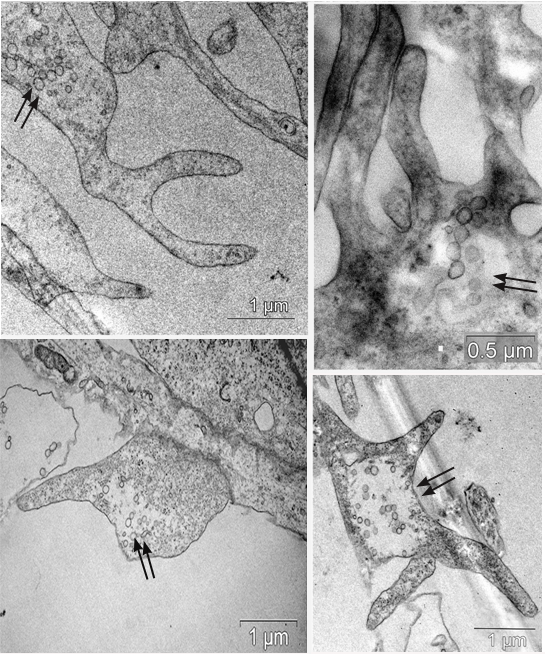

Supplement: Supplementary file 1 [file jpm-10-00215-s001.zip › FigS2.tif]
